# Structure, Luminescent Sensing and Proton Conduction of a Boiling-Water-Stable Zn(II) Metal-Organic Framework

**DOI:** 10.3390/molecules26165044

**Published:** 2021-08-20

**Authors:** Hua-Qun Zhou, Sai-Li Zheng, Can-Min Wu, Xin-He Ye, Wei-Ming Liao, Jun He

**Affiliations:** School of Chemical Engineering and Light Industry, Guangdong University of Technology, Guangzhou 510006, China; 3114001689@mail2.gdut.edu.cn (H.-Q.Z.); 1111906003@mail2.gdut.edu.cn (S.-L.Z.); 3217003865@mail2.gdut.edu.cn (C.-M.W.); 2111906026@mail2.gdut.edu.cn (X.-H.Y.)

**Keywords:** proton conduction, metal ion sensing, luminescence, metal-organic framework

## Abstract

A novel Zn(II) metal-organic framework [Zn_4_O(C_30_H_12_F_4_O_4_S_8_)_3_]_n_, namely **ZnBPD-4F4TS**, has been constructed from a fluoro- and thiophenethio-functionalized ligand 2,2′,5,5′-tetrafluoro-3,3′,6,6′-tetrakis(2-thiophenethio)-4,4′-biphenyl dicarboxylic acid (**H_2_BPD-4F4TS**). **ZnBPD-4F4TS** shows a broad green emission around 520 nm in solid state luminescence, with a Commission International De L’Eclairage (CIE) coordinate at x = 0.264, y = 0.403. Since d^10^-configured Zn(II) is electrochemically inert, its photoluminescence is likely ascribed to ligand-based luminescence which originates from the well-conjugated system of phenyl and thiophenethio moieties. Its luminescent intensities diminish to different extents when exposed to various metal ions, indicating its potential as an optical sensor for detecting metal ion species. Furthermore, **ZnBPD-4F4TS** and its NH_4_Br-loaded composite, **NH_4_Br@ZnBPD-4F4TS**, were used for proton conduction measurements in different relative humidity (RH) levels and temperatures. Original **ZnBPD-4F4TS** shows a low proton conductivity of 9.47 × 10^−10^ S cm^−1^ while **NH_4_Br@ZnBPD-4F4TS** shows a more than 25,000-fold enhanced value of 2.38 × 10^−^^5^ S cm^−1^ at 40 °C and 90% RH. Both of the proton transport processes in **ZnBPD-4F4TS** and **NH_4_Br@ZnBPD-4F4TS** belong to the Grotthuss mechanism with *E*_a_ = 0.40 and 0.32 eV, respectively.

## 1. Introduction

The luminescent metal-organic framework (LMOF) is a fascinating class of functional materials that has been extensively researched for its inspiring application in plentiful areas, especially chemical sensing of molecules and cation and anion species [1,2,3,4]. The luminescent and sensing property of LMOFs is related to original metal ions, functional ligands and self-assembly processes. To date, LMOF-based sensors have mainly focused on lanthanide-based (e.g., Eu^3+^, Tb^3+^) metal-organic frameworks largely because of their strong photoluminescence derived from the ligand-to-lanthanide antenna effect [5,6,7,8]. The high cost and almost unalterable emission wavelength of lanthanide LMOFs motivate researchers to develop LMOFs using transition metal ions (e.g., Zn^2+^, Cd^2+^, etc.) as metal nodes [9,10,11]. As some d^10^ metal ions (e.g., Zn^2+^) are proposed to exert less influence on the emission of LMOFs, ligand design is important for designing Zn-LMOF phosphors [12,13,14]. The introduction of an aromatic π-conjugated system into a ligand helps promote the red shift of the emission wavelength to the visible light region and improves the emission intensity. Further, aromatic groups are proposed to boost the interaction between the host framework and substrate [15,16]. Metal ion detection has been focused on extensively because excess use and emission of metal species have caused many issues, such as environmental pollution, health hazards, etc. [17,18]. Nevertheless, it is still a challenge to achieve LMOF-based sensors with high selectivity, sensitivity and recyclability. In particular, only a few examples of LMOF-based sensors of Mn^2+^ ions have been reported [19,20].

In recent years, MOF-based proton-conducting materials have received much attention due to their potential application in proton exchange membrane fuel cells [21,22,23,24]. Traditional Nafion materials have the shortcomings of high cost and intolerance of high temperature, which also accelerates the development of candidate materials [25]. MOFs can be constructed by commonly available metal ions and well-designed ligands, and many of them exhibit rather high solvent (i.e., H_2_O) and thermal (over 400 °C) stability [26,27,28]. This feature makes them an advantage in water-assisted proton conducting materials. Hydrated proton conduction is mainly determined by a hydrogen bond network created by appropriate porosity, functional groups on the ligand and proton carriers in the voids. This leads to two kinds of strategies for achieving high proton conductivity: (1) introducing acidic and hydrophilic groups including F, N, O and S atoms to the ligand [29,30,31,32,33]; (2) encapsulating guest molecules (e.g., water, imidazole, histamine, ammonium bromide, etc.) into the voids [34,35,36,37,38].

Herein, thiophenethio- and fluorine-functionalized linker molecule 2,2′,5,5′-tetrafluoro-3,3′,6,6′-tetrakis(2-thiophenethio)-4,4′-biphenyl dicarboxylic acid (**H_2_BPD-4F4TS**) was synthesized and used to react with Zn(NO_3_)_2_ through a solvothermal strategy, affording a metal-organic framework, **ZnBPD-4F4TS**. Photoluminescent measurement and metal ion sensing experiments for **ZnBPD-4F4TS** have been performed, showing metal ion-dependent quenching with luminescence intensity. The existence of pendant F- and S-included groups provide a platform for adjusting proton conduction. The NH_4_Br-loaded composite, **NH_4_Br@ZnBPD-4F4TS**, exhibits a highly improved conductivity (2.38 × 10^−5^ S cm^−1^) compared to that of original **ZnBPD-4F4TS** (9.47 × 10^−^^10^ S cm^−1^). It highlights encapsulating proton carriers into the framework as an effective strategy to enhance proton transport.

## 2. Results and Discussion

### 2.1. Synthesis and Structure Characterization

**Figure 1:**
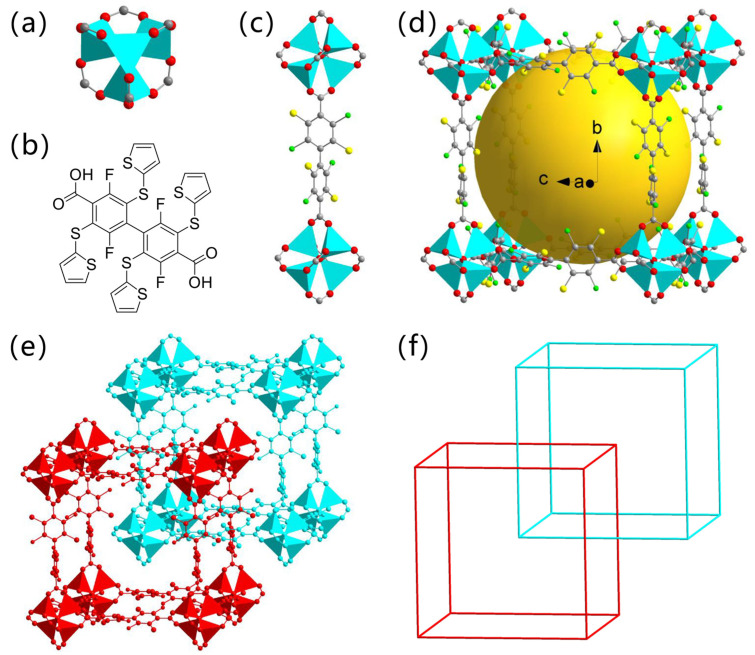
(**a**) Zn_4_O(COO)_6_ cluster of **ZnBPD-4F4TS**. (**b**) Ligand **H_2_BPD-4F4TS** used in this work. (**c**) Coordination mode diagram of linker BPD-4F4TS^2−^. (**d**) A three-dimensional network diagram of **ZnBPD-4F4TS**. The ball is present to display the inner void filled with disordered pendent thiophene groups. (**e**) Two-fold interpenetrated framework of **ZnBPD-4F4TS**. (**f**) A simplified topology of **ZnBPD-4F4TS**. Hydrogen atoms are omitted for clarity and thiophene groups are absent because of their high disorder. Atom color: red, oxygen; gray, carbon; green, fluorine; yellow, sulfur; cyan, Zn_4_O polyhedron.

Ligand **H_2_BPD-4F4TS**, 2,2′,5,5′-tetrafluoro-3,3′,6,6′-tetrakis(2-thiophenethio)-4,4′-biphenyl dicarboxylic acid, was synthesized according to our previous work [39]. Further self-assembly with zinc nitrate hexahydrate afforded light yellow square crystals (Figure S1), namely **ZnBPD-4F4TS**. The rigid framework of **ZnBPD-4F4TS** was well determined by single crystal X-ray diffraction (SCXRD) analysis (Figure 1). Unfortunately, the thiophene substituents were highly disordered and difficult to resolve. Therefore, **ZnBPD-4F4TS** was digested (in DCl/NaF/DMSO-*d*_6_) for ^1^H and ^1^^9^F NMR tests to verify the intact thiophene units of linker BPD-4F4TS^2−^ (Figures S2 and S3).

SCXRD results reveals that **ZnBPD-4F4TS** crystallizes in the cubic *I*23 space group with a = 17.232 Å and *α* = 90° (Table S1). The asymmetric unit is built from one crystallographically independent Zn^2+^ ion, one oxygen and 1/4 BPD-4F4TS^2−^ anion. The metal node consists of a Zn-O cluster-based secondary building unit (SBU) with a formula of Zn_4_O(COO)_6_ (Figure 1a). Each of these four Zn^2+^ ions is located at a tetrahedron center (cyan tetrahedron represents the ZnO_4_ polyhedron) and bridged by one μ_4_-O. Each Zn^2+^ ion bonds to four oxygen atoms in which one comes from a μ_4_-O and the other three are from three carboxylate groups of BPD-4F4TS^2−^. Each linear BPD-4F4TS^2−^ (Figure 1b) acts as a two-connected bridge to link the SBUs, forming a 3D framework (Figure 1c). Interconnected 1D channels partly filled by thiophene groups run along the *a*, *b* and *c* crystallographic axes (Figure 1d). The coordination environment of the Zn^2+^ ion and coordination mode of BPD-4F4TS^2−^ in **ZnBPD-4F4TS** are the same as previously reported IRMOF-type MOFs [40], but they show a two-fold interpenetrated aggregation (Figure 1d). The construction of SBUs and linkers can be simply reticulated into a **pcu** topology (Figure 1e).

**Figure 2:**
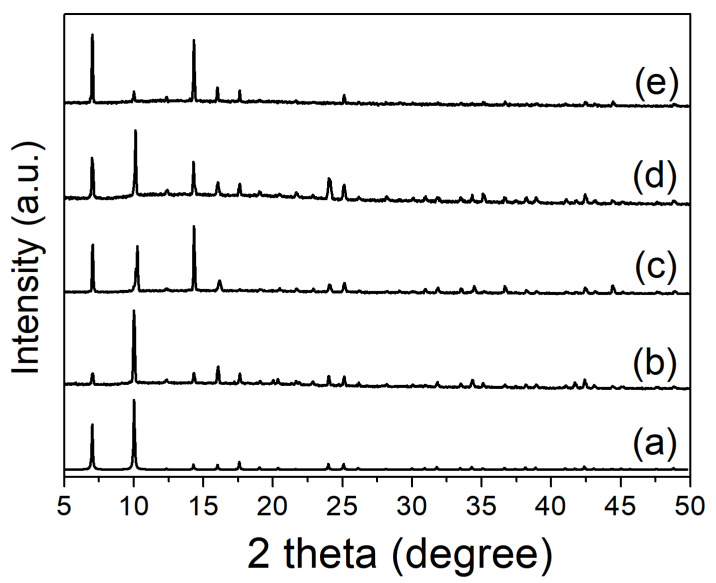
PXRD patterns of **ZnBPD-4F4TS**: (a) A simulated one obtained from single crystal data, (b) as-synthesized one, (c) after exposed to air for 21 days, (d) activated sample after acetone exchange and thermal treatment at 100 °C and vacuum, (e) after soaking in boiling water for 30 h.

Pure phase of as-synthesized **ZnBPD-4F4TS** was obtained with the solvothermal method according to a consistent powder X-ray diffraction (SCXRD) pattern with the simulated one, which was also verified by FT-IR spectra (Figure 2 and Figure S4). **NH_4_Br@ZnBPD-4F4TS** was prepared by immersing **ZnBPD-4F4TS** in a saturated NH_4_Br solution of ethanol at room temperature for 2 days. The loading amount of NH_4_Br in the voids was further determined to be 2.21 wt% by elemental analysis (EA) measurement.

### 2.2. Stability of **ZnBPD-4F4TS**

The stability of metal-organic frameworks is vital to their practical applications; therefore, the atmospheric, thermal and solvent stabilities have been investigated. **ZnBPD-4F4TS** was highly air and water stable judging from the unchanged PXRD patterns after exposure to the atmosphere for 21 days and soaking in boiling water for 30 h, respectively (Figure 2(c,e)). This excellent stability of **ZnBPD-4F4TS** to water is rare in reported Zn(II)-carboxylate frameworks [26,41]. As-synthesized crystalline **ZnBPD-4F4TS** was further Soxhlet-extracted by acetone and heated at 100 °C to prepare activated **ZnBPD-4F4TS**. EA data indicated a formula of [Zn_4_O(C_30_H_12_F_4_O_4_S_8_)_3_·(H_2_O)_1.5_(CH_3_CN)_0.6_]_n_ for activated **ZnBPD-4F4TS**. Consistent diffraction peaks with as-synthesized **ZnBPD-4F4TS** suggested an intact coordination framework (Figure 2(d)). The thermogravimetric (TG) curve of activated **ZnBPD-4F4TS** showed slight weight loss (3.15%) in the initial period, which could be attributed to the loss of a few water molecules from the air and remaining crystallized guest molecules in the pores. This result suggested the coordination framework was thermally stable below 380 °C (Figure S5). The subsequent large weight loss could be ascribed to decomposition of organic ligands and destruction of coordination bonds, which was supported by the endothermic effect in the differential thermal analysis (DTA) curve (Figure S5). The consistent PXRD pattern of **NH_4_Br@ZnBPD-4F4TS** with **ZnBPD-4F4TS** indicated that the host framework remained stable after immersion in NH_4_Br solution (Figure S6b).

### 2.3. Luminescent Properties

**Figure 3:**
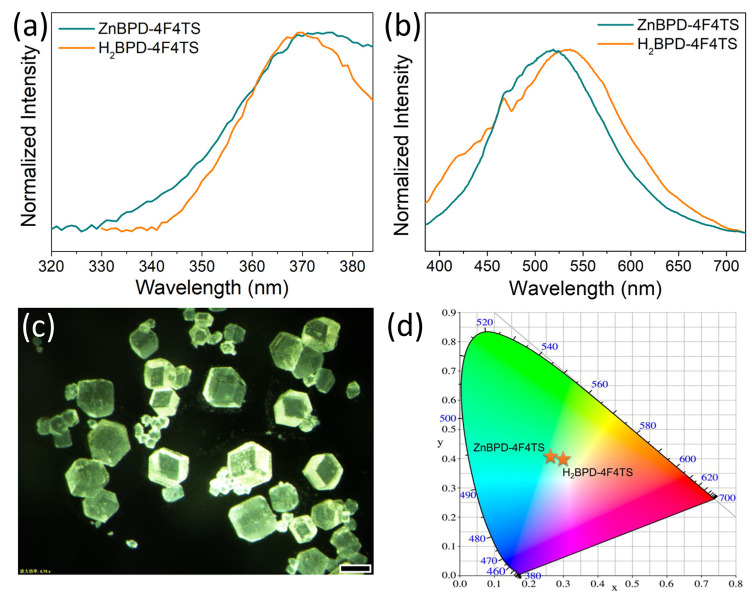
(**a**) Excitation spectra of ligand **H_2_BPD-4F4TS** (λ_em_ = 535 nm) and **ZnBPD-4F4TS** (λ_em_ = 518 nm) in the solid state. (**b**) Emission spectra of ligand **H_2_BPD-4F4TS** and **ZnBPD-4F4TS** when excited at 370 nm UV light in the solid state. (**c**) A photograph of as-synthesized **ZnBPD-4F4TS** single crystals under UV light irradiation. The scale in the figure is 100 μm. (**d**) CIE chromaticity diagram of ligand **H_2_BPD-4F4TS** and **ZnBPD-4F4TS** when excited at 370 nm.

With the conjugated thiophene and phenyl units, **ZnBPD-4F4TS** was expected to possess decent luminescent property. Both excitation and emission spectra of ligand **H_2_BPD-4F4TS** and **ZnBPD-4F4TS** were recorded in the solid state (Figure 3a,b). In the case of ligand **H_2_BPD-4F4TS**, it displays one fluorescent emission band centered at 535 nm when excited at 370 nm, which is probably assigned to π or n to π* orbital transitions [42,43]. After coordination to form **ZnBPD-4F4TS**, it shows a similar but blue-shifted emission peak at 518 nm when excited at 370 nm, showing a green emission of crystals (Figure 3c). This blue shift of **ZnBPD-4F4TS** in comparison to free ligand is probably attributed to the metal-ligand coordination interaction and deprotonated effect of the dicarboxylic acid [44,45]. Accordingly, Commission International De L’Éclairage (CIE) coordinates change from (0.297, 0.396) for the ligand to (0.264, 0.403) for **ZnBPD-4F4TS** (Figure 3d). In addition, the emission spectrum of **NH_4_Br@ZnBPD-4F4TS** was also obtained and found to be quite similar to that of as-synthesized **ZnBPD-4F4TS** (Figure S7).

### 2.4. Metal Sensing and Mechanism

**Figure 4:**
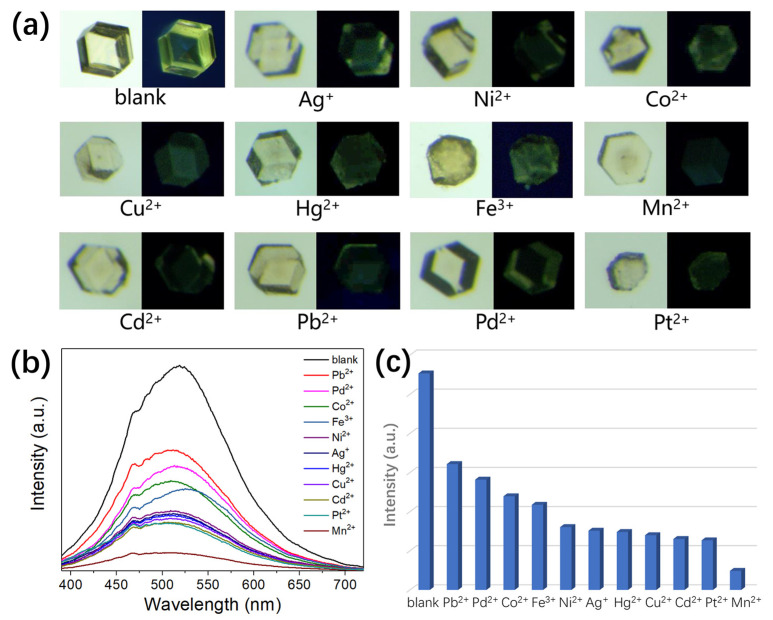
(**a**) Photographs of **ZnBPD-4F4TS** crystals under natural light and 365 nm UV light irradiation after immersion in water and aqueous solution of different metal ions (500 ppm). (**b**) Room temperature emission spectra (λ_ex_ = 370 nm) of **ZnBPD-4F4TS** after immersion in water and aqueous solution of different metal ions (500 ppm). (**c**) Emission intensity data at 518 nm according to the spectra from (**b**).

Excellent water stability, free-standing thiophenethio-functions in the pores and potential accessible pores inspired us to investigate the chemical sensing performance of **ZnBPD-4F4TS** in aqueous media. As-synthesized crystals of **ZnBPD-4F4TS** were first immersed in 500 ppm (based on metal species) metal chloride or nitrate solutions (Pb^2+^, Pd^2+^, Co^2+^, Fe^3+^, Ni^2+^, Hg^2+^, Cu^2+^, Cd^2+^, Pt^2+^, Mn^2+^ and Ag^+^) for 2 h at 80 °C and then selected for photography (Figure 4a). All of these crystals show no obvious color change, though some exhibit a semi-transparent appearance. When irradiated under 365 nm UV light, these crystals exhibit similar emission colors but with variously decreased brightness in comparison to as-synthesized **ZnBPD-4F4TS**. Emission spectra of bulk samples were also measured in the wavelength range of 380 to 720 nm, and they show different extents of reduction in luminescence intensity (monitored at 518 nm) after immersion in various metal ion solutions (Figure 4b). In particular, the case of Mn^2+^ exhibits the strongest luminescence quenching (Figure 4c). PXRD patterns of **ZnBPD-4F4TS** were found to be unchanged after exposure to various metal ion solutions, suggesting an intact framework structure (Figure S8).

### 2.5. Proton Conduction

**Figure 5:**
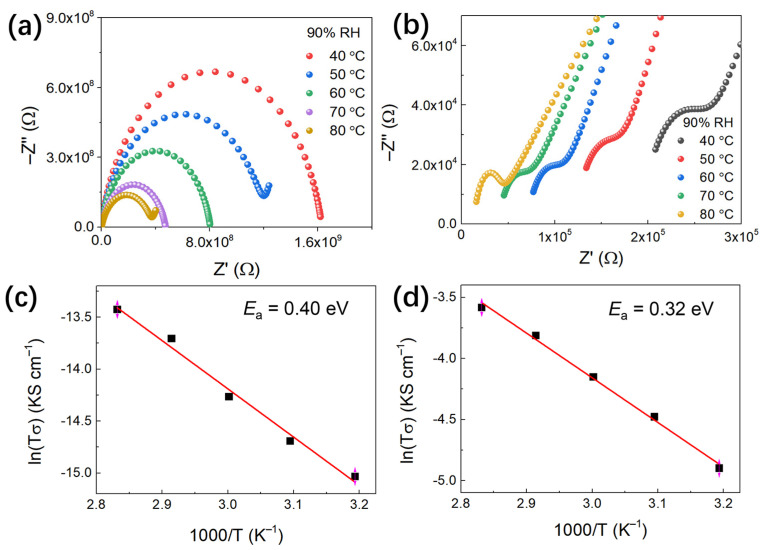
Nyquist plots of (**a**) **ZnBPD-4F4TS** and (**b**) **NH_4_Br@ZnBPD-4F4TS** at different temperatures (from 40 to 80 °C) and 90% RH. Dependence of proton conductivity in (**c**) **ZnBPD-4F4TS** and (**d**) **NH_4_Br@ZnBPD-4F4TS** as a function of temperature at 90% RH.

The proton carrier is one of the vital factors for improving proton conductivity. This motivated us to optimize the as-synthesized framework by encapsulating protonic guests (i.e., NH_4_Br) into framework voids through a post-synthetic strategy. After **ZnBPD-4F4TS** was immersed in NH_4_Br/EtOH solution for 2 days, **NH_4_Br@ZnBPD-4F4TS** was separated, washed and dried. The ac impedance measurements were carried out with compacted pellets of **ZnBPD-4F4TS** and **NH_4_Br@ZnBPD-4F4TS**, respectively. The corresponding Nyquist plots are shown at different temperatures at 90% relative humidity (RH) (Figure 5a,c). In a typical measurement, the proton conductivity of a sample is determined by the high frequency region with the following equation:

*σ* = *l*/*RS* (1)

where *l* is the thickness (mm) and *S* is the cross-sectional area (mm^2^) of the pellet, while *R* (Ω) can be calculated from the Nyquist impedance plots [46,47]. Accordingly, the corresponding proton conductivity was obtained and is listed in Table S2. It is exciting that **NH_4_Br@ZnBPD-4F4TS** (2.38 × 10^−^^5^ S·cm^−1^) exhibits a 25,000-fold increased proton conductivity compared to the original **ZnBPD-4F4TS** (9.47 × 10^−10^ S·cm^−1^) at 40 °C and 90% RH. This high and sharply increased proton conductivity of **NH_4_Br@ZnBPD-4F4TS** highlights that manipulation of protonic guests serves as an effective strategy to promote proton transport in the framework. Further research reveals that **ZnBPD-4F4TS** possesses increasing proton conductivity varying from 9.47 × 10^−10^ (40 °C) to 4.19 × 10^−9^ (80 °C) S·cm^−1^ when the temperature increases under 90% RH (Figure S9). Similarly, the conductivity of **NH_4_Br@ZnBPD-4F4TS** increases from to 2.38 × 10^−^^5^ (40 °C) to 7.87 × 10^−^^5^ (80 °C) S·cm^−1^ (Figure S9). Both of the metal-organic frameworks remain stable after proton conduction measurements according to the unchanged PXRD patterns (Figures S6 and S10).

### 2.6. Proton Conduction Mechanism

The obtained conductivities of **ZnBPD-4F4TS** and **NH_4_Br@ZnBPD-4F4TS** at various temperatures are plotted against temperature in the form of ln(*σT*) against 1000/*T* (Figure 5b,d), and the activation energy (*E*_a_) of proton transport can be estimated using the Arrhenius equation:

*σT* = *σ*_0_*exp*(−*E_a_*/*kT*) (2)

The *E_a_* value is calculated to be 0.40 and 0.32 eV for **ZnBPD-4F4TS** and **NH_4_Br@ZnBPD-4F4TS**, respectively. Generally, the proton transport process is classified into Grotthuss [48] (*E_a_* < 0.4 eV) and vehicle [49] (*E_a_* > 0.4 eV) mechanisms according to the *E_a_* value. Therefore, both of these materials can be attributed to the Grotthuss mechanism. It indicates that proton transport is achievable through the hydrogen bond network of proton carriers. Both of the H_2_O molecules and electronegative S atoms in the framework help form the hydrogen bonds to promote proton transport. **NH_4_Br@ZnBPD-4F4TS** shows a 25,000-fold improvement in conductivity compared to **ZnBPD-4F4TS** that might originate from the more favorable hydrogen bond channels among H_2_O, thiophene and NH_4_Br species.

## 3. Materials and Methods

### 3.1. General Procedure

Starting materials, reagents and solvents were purchased from commercial sources (J&K, Aldrich and Acros) and used without further purification. Elemental analysis (EA) was performed with a Vario Micro CUBE CHN elemental analyzer (Elementar, Germany). FT-IR spectra were obtained using a Avatar 360 spectrophotometer (Thermo Nicolet, The United States). Nuclear magnetic resonance (NMR) spectra were recorded at 298 K on a 400 MHz superconducting magnet high-field NMR spectrometer (Bruker, The Swiss), with working frequencies of 400 MHz for ^1^H, 376 MHz for ^19^F. Chemical shifts are reported in ppm relative to the signals corresponding to the residual non-deuterated solvents, with tetramethylsilane (TMS) as the internal standard. Thermogravimetric (TG) analyses were carried out in a nitrogen stream using Thermal analysis equipment (STA 6000) (PerkinElmer, The United States) with a heating rate of 10 °C/min. Powder X-ray diffraction (PXRD) data were collected in reflection mode at room temperature on a Smart Lab diffractometer (Rigaku, Japan) with a mixture of Cu-Kα1 (λ = 1.54056 Å) and Cu-Kα2 (λ = 1.5418 Å) radiation. Fluorescence spectra were measured on a FluoroMax-4 fluorescence spectrometer (HORIBA Jobin Yvon, France) at room temperature.

### 3.2. Synthesis of [Zn_4_O(H_2_BPD-4F4TS)_3_]_n_ (**ZnBPD-4F4TS**)

The ligand **H_2_BPD-4F4TS** (5 mg, 0.0065 mmol) and zinc nitrate hexahydrate (5 mg, 0.017 mmol) were weighed into a glass tube 8 mm in diameter, and then a mixed solvent of water and acetonitrile (0.8 mL, *v*/*v* = 1:1) was added. The mixture was sonicated for 5 min to form a clear solution. Then, the glass tube nozzle was melted and sealed at high temperature, and the glass tube was heated at 140 °C for 36 h, followed by natural cooling to room temperature, during which light yellow truncated cube-like crystals (2.4 mg, 14% based on **H_2_BPD-4F4TS**) were formed.

### 3.3. Activation of **ZnBPD-4F4TS**

To exchange and remove the solvent molecules from the pores of **ZnBPD-4F4TS**, a thimble (e.g., made from folding filter paper) containing as-synthesized **ZnBPD-4F4TS** crystals (50 mg) was loaded into the main chamber of a Soxhlet extractor. The Soxhlet extractor was connected to a 250 mL round-bottomed flask including acetone (150 mL) and a magnetic stirring bar, and then equipped with a water condenser. The flask was heated to 100 °C with an oil bath for 3 days. The filter paper was then taken out and the solid was heated at 90 °C under vacuum to give the activated **ZnBPD-4F4TS** sample. Elemental analysis found [C (41.35%), H (1.57%), S (29.81%), N (0.33%)], a fitting formula can be determined to be Zn_4_O(C_30_H_12_F_4_O_4_S_8_)_3_(H_2_O)_1.5_(CH_3_CN)_0.6_ (m.w. 2635.86), which gives a calculated profile as [C (41.56%), H (1.56%), S (29.19%), N (0.32%)]. FT-IR (KBr pellet, ν/cm^−1^): 3444 (w), 1622 (s), 1434 (s), 1398 (s), 1385 (s), 1363 (s), 1218 (m), 1143 (w), 1103 (w), 986 (m), 907 (m), 850 (m), 803 (m), 767 (w), 741 (m), 698 (s), 623 (w), 584 (w), 517 (w), 477 (w), 447 (w).

### 3.4. Synthesis of **NH_4_Br@ZnBPD-4F4TS**

The solid sample of as-synthesized **ZnBPD-4F4TS** (30 mg) was added to a small glass bottle containing 4 mL of saturated ammonium bromide in ethanol solution, and soaked at room temperature for 2 days. Afterwards, the resultant solid **NH_4_Br@ZnBPD-4F4TS** was isolated by centrifugation and then washed with ethanol and acetone three times, and dried under vacuum for 1 h. Elemental analysis found [C (40.99%), H (1.52%), S (28.66%), N (0.37%)], a fitting formula can be determined to be Zn_4_O(C_30_H_12_F_4_O_4_S_8_)_3_(H_2_O)_0.5_(NH_4_Br)_0.6_(CH_3_CN)_0.1_ (m.w. 2656.08), which gives a calculated profile as [C (40.79%), H (1.51%), S (28.97%), N (0.37%)].

### 3.5. Single Crystal X-ray Crystallography

Single crystal data for **ZnBPD-4F4TS** were collected using a Bruker APEX-II CCD diffractometer (Bruker, Germany) with an I-mu-S micro-focus X-ray source using Cu Kα radiation (λ = 1.54178). Data were collected at 230.0 K. Reflections were indexed and processed, and the files scaled and corrected for absorption using APEX3 v2018. The space group was assigned and the structure was solved by direct methods using XPREP-2014/2 program and refined by full matrix least squares against *F*^2^ with all reflections using Shelxl2018 using the graphical interface Olex2 [50]. All non-hydrogen atoms were refined with anisotropic thermal parameters, and all hydrogen atoms were included in calculated positions and refined with isotropic thermal parameters riding on those of the parent atoms. The hanging thiophene groups are highly disordered and difficult to resolve. Therefore, their electron peaks were squeezed in the refinement process. The crystallographic data for incomplete **ZnBPD-4F4TS**, in CIF format, have been deposited with the Cambridge Crystallographic Data Centre as CCDC 2098157. These data can be obtained free of charge from the Cambridge Crystallographic Data Centre via www.ccdc.cam.ac.uk/data_request/cif (accessed on 22 July 2021).

### 3.6. Metal Ion Sensing Experiment

**ZnBPD-4F4TS** crystals (2 mg) were first introduced into different metal ion aqueous solutions (3 mL, 500 ppm based on metal ion) of MCl_x_ (M^x+^ = Hg^2+^, Cd^2+^, Ni^2+^, Co^2+^, Mn^2+^, Cu^2+^, Pt^2+^, Pd^2+^, Fe^3+^, Pb^2+^), AgNO_3_ and then heated at 80 °C for 2h. After cooling to room temperature, the bulk samples were centrifuged and washed three times with water. Additionally, they were washed three times with acetone and dried under vacuum. The dried samples were then used for luminescence measurements and the luminescence data were collected. The blank sample was obtained from **ZnBPD-4F4TS** crystals being immersed in pure water instead of metal ion solution.

### 3.7. Electrochemical Impedance Spectroscopy

The Nyquist plots (Z″ vs. Z′) of proton-conducting MOF often show a single semicircle at high frequency, representing proton resistivity contributions of the bulk sample. The proton conductivity was deduced from the semicircle by fitting an equivalent circuit which consists of Rs, R1 and W1 in the frequency range from 10 MHz to 1 Hz. Rs corresponds to wire and electrode resistance, R1 is proton resistance and W1 is the resistivity of the grain boundary. Sometimes W1 is not necessary, because the impedance plot of the capacitive tail may not appear in the measured range due to the high magnitude of the resistivity. The water-assisted conductivities of synthesized materials were measured under different relative humidity and temperature conditions and were further fitted with different fitting circuits using the ZView software [51].

## 4. Conclusions

In summary, a green emitter (x = 0.264, y = 0.403), **ZnBPD-4F4TS**, has been constructed from a fluorine- and thiophenethio-functionalized ligand. Exposed to various metal ions, it exhibits different reductions in luminescent intensity. In particular, the luminescence is almost fully quenched when exposed to Mn^2+^ ions. Moreover, NH_4_Br-loaded **NH_4_Br@ZnBPD-4F4TS** shows a more than 25,000-fold enhanced proton conductivity compared to the original **ZnBPD-4F4TS** at 40 °C and 90% RH, serving as an example of the enhancement of proton conducting material by post-synthetic modification.

## Data Availability

All data are included in the article.

## Supplementary Materials

The following are available online. Figure S1: A photograph of as-synthesized **ZnBPD-4F4TS** single crystals. Figure S2: Solution ^1^H NMR spectra of the activated sample of **ZnBPD-4F4TS** dissolved in DCl (38 % in D_2_O)/DMSO-*d*_6_ (v:v = 1:4) solution. Figure S3: Solution ^19^F NMR spectra of the activated sample of **ZnBPD-4F4TS** dissolved in DCl (38 % in D_2_O)/DMSO-*d*_6_ (v:v = 1:4) solution. Figure S4: FT-IR spectra of (a) the ligand **H_2_BPD-4F4TS** and (b) as-made **ZnBPD-4F4TS**; (c) **ZnBPD-4F4TS** after exposed to air for 21 days; (d) **ZnBPD-4F4TS** after soaking in boiling water for 30 h. Figure S5: Thermogravimetric plots of activated **ZnBPD-4F4TS**. Figure S6: PXRD patterns of (a) simulation from single crystal of **ZnBPD-4F4TS**; (b) as-synthesized **NH_4_Br@ZnBPD-4F4TS**; (c) **NH_4_Br@ZnBPD-4F4TS** after proton conduction test. Figure S7: Room temperature emission spectra of crystals **NH_4_Br@Zn-4F4TS** in the solid state (λ_ex_=370 nm). Figure S8: PXRD patterns of **ZnBPD-4F4TS** after immersion in various metal ion solutiona. Figure S9: Proton conductivities of **ZnBPD-4F4TS** and **NH4Br@ZnBPD-4F4TS** at different temperatures (from 40 °C to 80 °C) and 90% RH. Figure S10: PXRD patterns of (a) simulation from single crystal of **ZnBPD-4F4TS**; (b) as-synthesized **ZnBPD-4F4TS**; (c) **ZnBPD-4F4TS** after proton conduction test. Table S1: Crystallographic refinement parameters and results of **ZnBPD-4F4TS**.

## Sample Availability

Samples of the compounds are not available from the authors.
