# Supplementary material for: Structure, Luminescent Sensing and Proton Conduction of a Boiling-Water-Stable Zn(II) Metal-Organic Framework
Source: Molecules. 2021 Aug 20;26(16):5044. doi: 10.3390/molecules26165044 (PMC8401761; doi:10.3390/molecules26165044)
Supplement: Supplementary file 1 [file molecules-26-05044-s001.zip › molecules-1332290-supplementary.pdf]

Supporting information

Structure, Luminescent Sensing and Proton Conduction of a Boiling-Water-Stable Zn(II) Metal-Organic Framework

Hua-Qun Zhou †, Sai-Li Zheng †, Can-Min Wu, Xin-He Ye, Wei-Ming Liao* and Jun He*

School of Chemical Engineering and Light Industry, Guangdong University of Technology, Guangzhou 510006, China; 3114001689@mail2.gdut.edu.cn (H.-Q.Z.); 1111906003@mail2.gdut.edu.cn (S.-L.Z.); 3217003865@mail2.gdut.edu.cn (C.-M.W.); 2111906026@mail2.gdut.edu.cn (X.-H.Y.)

* Correspondence: wmliao@gdut.edu.cn (W.-M.L.); junhe@gdut.edu.cn (J.H.)

† These authors contributed equally to this work.

Figures and Tables

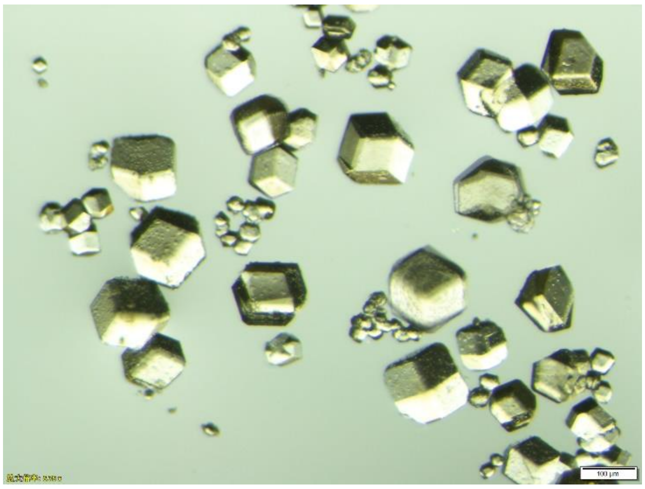

Figure S1 A photograph of as-synthesized **ZnBPD-4F4TS** single crystals. The scale in the figure is 100 μm.

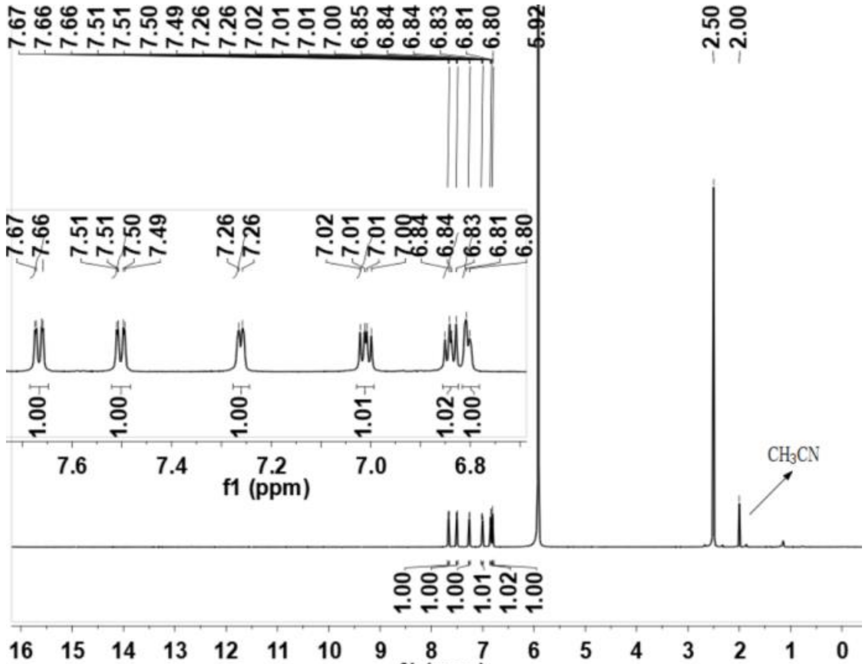

Figure S2 Solution ^{1}H NMR spectra of the activated sample of **ZnBPD-4F4TS** dissolved in DCl (38 % in D_2O)/DMSO-d_6 (v:v = 1:4) solution.

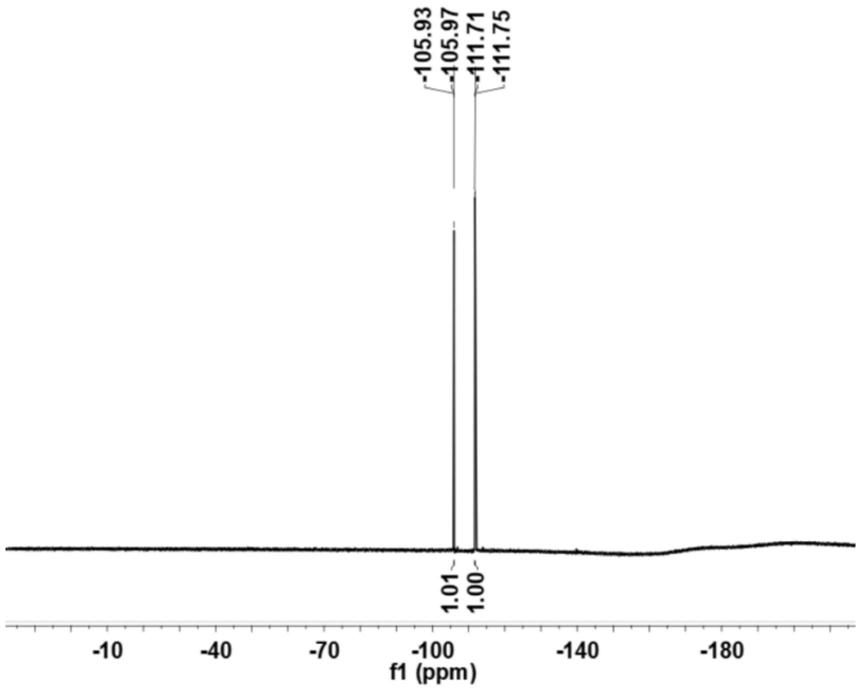

Figure S3 Solution ^{19}F NMR spectra of the activated sample of **ZnBPD-4F4TS** dissolved in DCl (38 % in D_2O)/DMSO-d_6 (v:v = 1:4) solution.

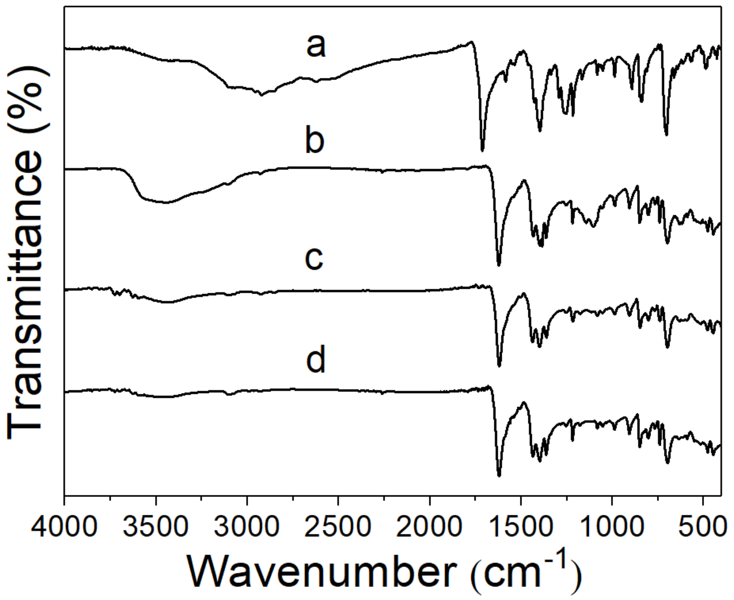

Figure S4 FT-IR spectra of (a) the ligand **H_2BPD-4F4TS** and (b) as-made **ZnBPD-4F4TS**; (c) **ZnBPD-4F4TS** after staying in air for 21 days; (d) **ZnBPD-4F4TS** after soaking in boiling water for 30 h.

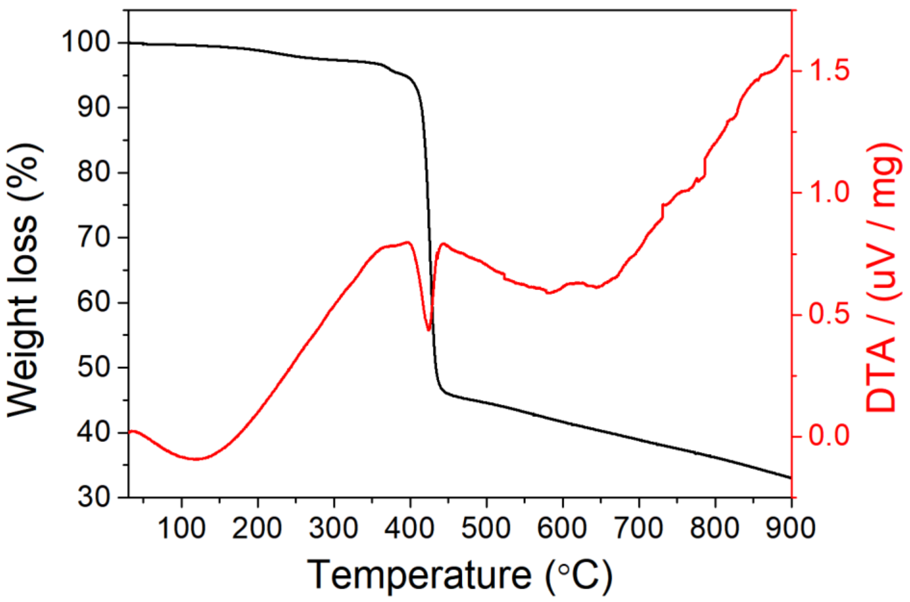

Figure S5 Thermogravimetric plots and differential thermal analysis (DTA) curve of activated **ZnBPD-4F4TS**.

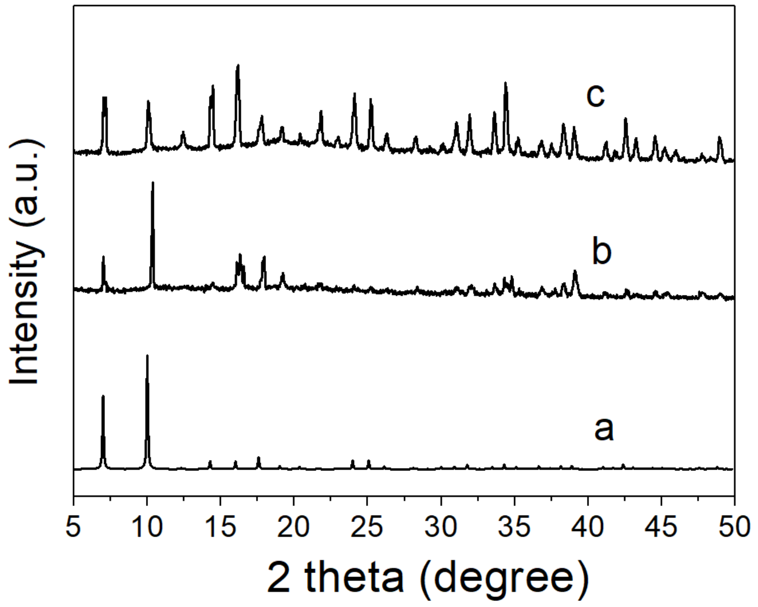

Figure S6 PXRD patterns of (a) simulation from single crystal of **ZnBPD-4F4TS**; (b) as-synthesized **NH_4Br@ZnBPD-4F4TS**; (c) **NH_4Br@ZnBPD-4F4TS** after proton conduction test.

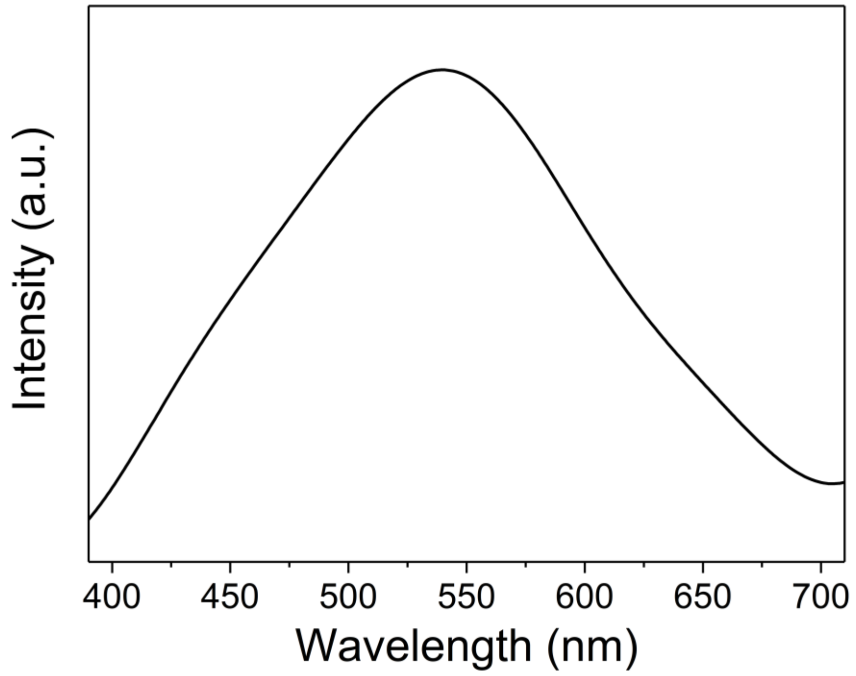

Figure S7 Room temperature emission spectra of crystals **NH_4Br@Zn-4F4TS** in the solid state (λ_{ex}=370 nm).

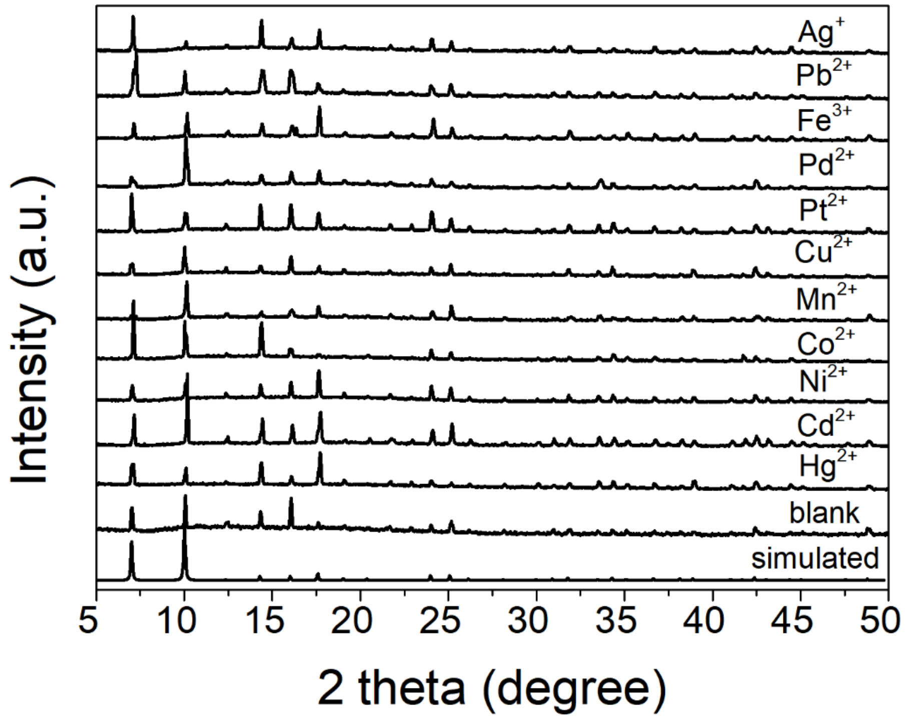

Figure S8 PXRD patterns of **ZnBPD-4F4TS** after immersing in various metal ion solution.

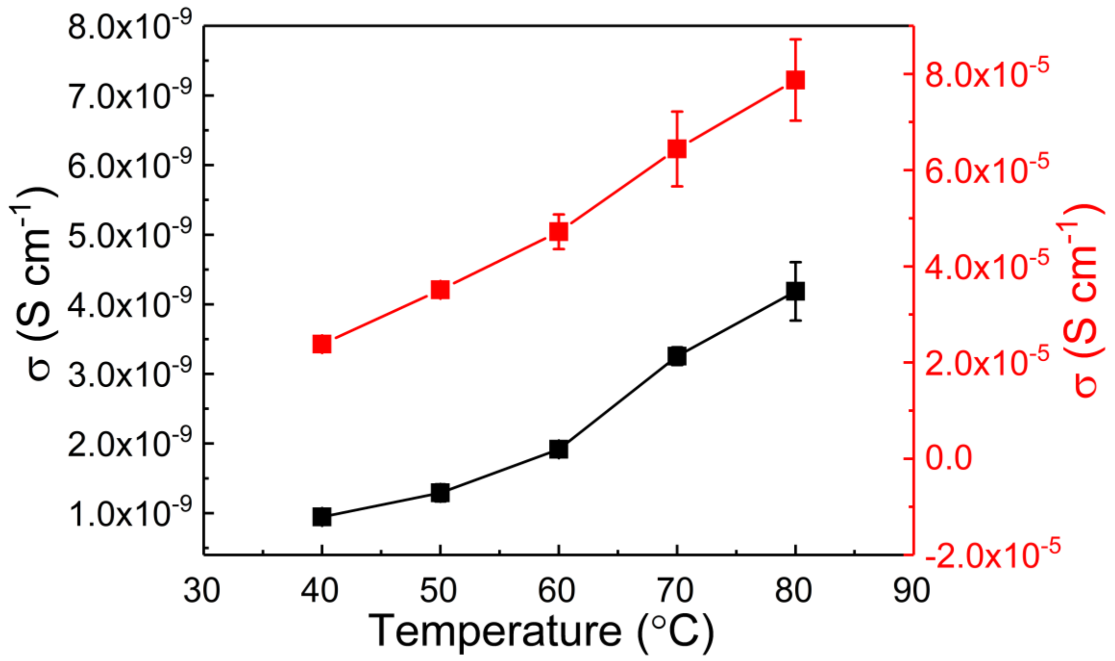

Figure S9 Proton conductivities of **ZnBPD-4F4TS** and **NH_4Br@ZnBPD-4F4TS** at different temperatures (from 40 °C to 80 °C) and 90% RH.

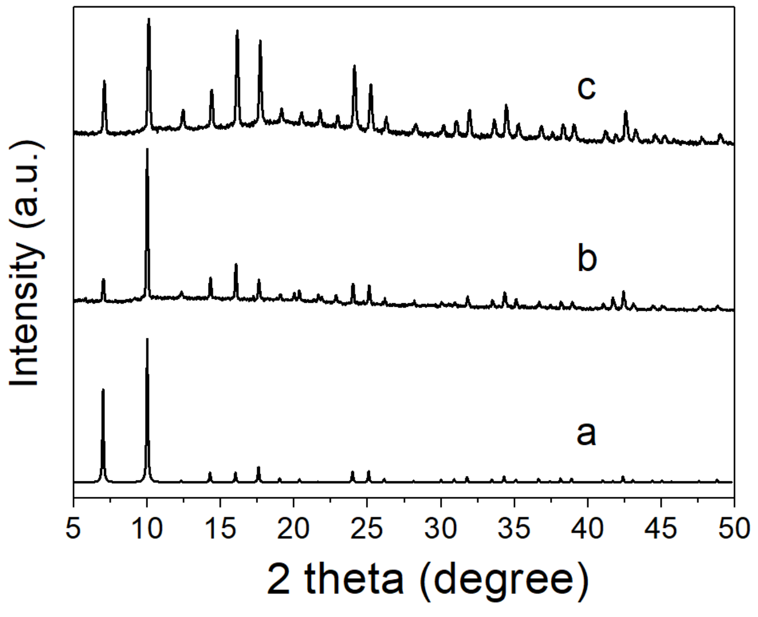

Figure S10 PXRD patterns of (a) simulation from single crystal of **ZnBPD-4F4TS**; (b) as-synthesized **ZnBPD-4F4TS**; (c) **ZnBPD-4F4TS** after proton conduction test.

Table S1 Crystallographic refinement parameters and results of **ZnBPD-4F4TS** without hanging thiophene groups.

Compound	**ZnBPD-4F4TS**
Empirical formula	$C_{42}F_{12}O_{13}S_{12}Zn_4$
Formula weight	1586.70
Temperature/K	230.0
Crystal system	cubic
Space group	$I23$
a/Å	17.2315(5)
b/Å	17.2315(5)
c/Å	17.2315(5)
α/°	90.00
β/°	90.00
γ/°	90.00
Volume/Å^3	5116.5(4)
Z	2
Dc/g·cm^{-3}	1.030
μ/mm^{-1}	3.850
F (000)	1552.00
R_1[a] (I>=2σ (I))	0.0875
wR_2[b] (all data)	0.2465
GOOF	1.073

[a] $R_1=\sum(||F_0|-|F_c||)/\sum|F_0|$; [b] $wR_2=(\sum w(F_0^2-F_c^2)^2/\sum w(F_0^2)^2)^{1/2}$
